# HyMNet: A Multimodal Deep Learning System for Hypertension Prediction Using Fundus Images and Cardiometabolic Risk Factors

**DOI:** 10.3390/bioengineering11111080

**Published:** 2024-10-29

**Authors:** Mohammed Baharoon, Hessa Almatar, Reema Alduhayan, Tariq Aldebasi, Badr Alahmadi, Yahya Bokhari, Mohammed Alawad, Ahmed Almazroa, Abdulrhman Aljouie

**Affiliations:** 1 AI and Bioinformatics Department, King Abdullah International Medical Research Center, King Saud bin Abdulaziz University for Health Sciences (KSAU-HS), Riyadh 11481, Saudi Arabia; mohammedsalimab@outlook.com (M.B.); almatarh@kaimrc.edu.sa (H.A.); alduhayanr@kaimrc.edu.sa (R.A.); bokhariy@kaimrc.edu.sa (Y.B.); 2 Data Management Department, King Abdullah International Medical Research Center, King Saud bin Abdulaziz University for Health Sciences (KSAU-HS), Riyadh 11481, Saudi Arabia; 3 Ophthalmology Department, King Abdulaziz Medical City, Ministry of National Guard Health Affairs, Riyadh 14611, Saudi Arabia; debasit@ngha.med.sa; 4 Ophthalmology Department, Prince Mohammad bin Abdulaziz Hospital, Ministry of National Guard Health Affairs, Al Madinah 42324, Saudi Arabia; ba25dr@gmail.com; 5 College of Public Health and Health Informatics, King Saud bin Abdulaziz University for Health Sciences (KSAU-HS), Riyadh 14815, Saudi Arabia; 6 National Center for Artificial Intelligence (NCAI), Saudi Data and Artificial Intelligence Authority (SDAIA), Riyadh 12382, Saudi Arabia; malawad@sdaia.gov.sa

**Keywords:** artificial intelligence, machine learning, computer vision, cardiovascular diseases, hypertension detection, fundus images, multimodal deep learning

## Abstract

Study Objectives: This study aimed to develop a multimodal deep learning (MMDL) system called HyMNet, integrating fundus images and cardiometabolic factors (age and sex) to enhance hypertension (HTN) detection. Methods: HyMNet employed RETFound, a model pretrained on 1.6 million retinal images, for the fundus data, in conjunction with a fully connected neural network for age and sex. The two pathways were jointly trained by joining their feature vectors into a fusion network. The system was trained on 5016 retinal images from 1243 individuals provided by the Saudi Ministry of National Guard Health Affairs. The influence of diabetes on HTN detection was also assessed. Results: HyMNet surpassed the unimodal system, achieving an F1 score of 0.771 compared to 0.745 for the unimodal model. For diabetic patients, the F1 score was 0.796, while it was 0.466 for non-diabetic patients. Conclusions: HyMNet exhibited superior performance relative to unimodal approaches, with an F1 score of 0.771 for HyMNet compared to 0.752 for models trained on demographic data alone, underscoring the advantages of MMDL systems in HTN detection. The findings indicate that diabetes significantly impacts HTN prediction, enhancing detection accuracy among diabetic patients. Utilizing MMDL with diverse data sources could improve clinical applicability and generalization.

## 1. Introduction

Cardiovascular diseases (CVDs) are a primary cause of mortalities worldwide, making up around a third of all global deaths in 2021, with hypertension (HTN) or high blood pressure (BP) serving as significant risk factors [1,2,3]. High BP is responsible for approximately 54% of stroke cases and 47% of coronary heart disease incidents globally [4]. Moreover, it increases the likelihood of developing HTN-mediated organ damage such as retinopathy and renal failure [5,6]. Despite the severe and life-threatening consequences of HTN, nearly 46% of adults with high BP remain unaware of their condition [7]. Consequently, there is a pressing need for tools that can facilitate the early detection and identification of HTN, which can aid in CVD-risk stratification and prevent further complications [8].

Current HTN screening criteria rely on BP measurements. Outpatient BP measurements may not accurately represent the true BP of a patient, leading to potential over- or underestimations, which can subsequently affect appropriate CVD risk stratification [9,10,11,12]. A single instance of a high BP reading in a clinical setting might merely be a manifestation of white coat syndrome and not indicative of the actual chronic BP of an individual [13,14,15]. Conversely, a normal BP measurement could provide false reassurance and mask underlying HTN [15,16,17]. Because HTN can affect microvascular structures in its early onset, considerably before manifesting clinical signs and symptoms, microvascular health assessments may offer a more accurate representation of CVD risks in outpatient settings [18,19].

Thus, high BP can cause microvascular damage in its early stages, causing alterations in small blood vessels, such as narrowing and ruptures [20]. Specifically, high BP can initially result in focal arteriolar narrowing and arteriovenous nipping within the retina, progressing to lipid exudation (visible as hard exudates) and ischemia of the nerve fiber layers (cotton wool spots) in advanced or more severe stages [18]. The retina is unique in that it allows non-invasive visualization of the vasculature [21,22]. Consequently, retinal fundus imaging can capture vascular changes induced by HTN, making it a promising method for early HTN detection and screening [22,23,24].

Deep learning (DL) is a subset of machine learning algorithms designed to automatically learn and extract complex patterns and features from vast amounts of data [25]. Thus, these algorithms can capture subtle details and relationships within fundus images that may not be immediately apparent to the human eye, thereby providing better detection ability for microvascular changes. By effectively processing and analyzing these intricate visual cues, DL models can potentially outperform traditional diagnostic methods and offer more accurate and efficient HTN detection, especially in its early stages. In the past, DL models have demonstrated exceptional performance for classifying retinal diseases such as diabetic retinopathy, hypertensive retinopathy, and glaucoma from fundus photographs [26,27,28,29].

Multimodal DL (MMDL) involves the use of heterogeneous data modalities to train DL systems. Similar to physicians, whose decisions are based on inputs from various sources, such as physical examinations, patient history, and laboratory results, DL models must also incorporate data from multiple modalities to achieve clinician-level accuracy. Recent studies have shown that MMDL can improve the predictive performance for CVD risk assessments and detection by utilizing diverse data inputs [30,31,32].

Previous studies analyzed the feasibility of predicting hypertensive retinopathy from fundus images [33,34,35,36,37,38,39]. Cen et al. [38] developed neural networks that could detect 39 different fundus diseases and conditions, achieving an AUC score of 0.99 for predicting severe hypertensive retinopathy from fundus images, on a multihospital test set of 60,445 images. In addition, Bhimavarapu et al. [33] trained a support vector machine on 1200 fundus images to classify cases into five hypertensive retinopathy severity levels, achieving an accuracy of 98.9%. Lastly, Qureshi et al. [39] used 9500 retinal images to train and evaluate a depth-wise separable convolutional neural network, achieving an AUC of 0.96.

Moreover, studies have explored the application of DL to systemic HTN classification using fundus photographs, which is a more difficult task. Zhang et al. [40] utilized an Inception-v3 network to classify individuals with HTN (defined as those with systolic BP > 140 mmHg or diastolic BP > 90 mmHg) from those with normal BP. They evaluated their proposed model on a dataset comprising 1222 fundus images obtained from a population in Central China and reported an area under the receiver operating characteristic (ROC) curve (AUC) of 0.766 for HTN classification. Poplin et al. [41] demonstrated the potential of DL for extracting valuable cardiometabolic risk factors from retinal images using an Inception-v3 architecture-based model trained on 1,779,020 images obtained from EyePACS and the UK Biobank dataset. The model accurately extracted the age of patients with a mean absolute error (MAE) of 3.26 years, sex with an AUC of 0.97, smoking status with an AUC of 0.71, systolic and diastolic BPs with MAE values of ±11.35 and ±6.42 mmHg, respectively, and body mass index with an MAE of ±3.29. Building on this study, Gerrits et al. [42] investigated the potential mediating effects of age and sex on the predictive performance of MobileNet-V2, a DL architecture, for cardiometabolic risk factors such as HTN, using a dataset of 12,000 fundus photographs from the Qatar Biobank. Their findings indicated that age and sex could act as mediating variables for predicting BP and other cardiometabolic risk factors.

This study expands upon the work of Zhang et al. [40] and Gerrits et al. [42] by proposing an MMDL system that integrates fundus photographs with cardiometabolic risk factors using various data fusion techniques. Age and sex were selected as supplementary features owing to their ease of accessibility and considerable roles as risk factors for HTN. Therefore, the proposed MMDL system has the potential to assess HTN risks in ophthalmological clinics and can serve as a valuable screening tool for early HTN detection.

We summarized our contributions as the following:

- We developed HyMNet, a multimodal deep learning system that combines fundus images with demographic features to improve hypertension detection capabilities.
- We investigated the effects underlying diabetes has on HyMNet’s predictive abilities and concluded that HyMNet strongly relies on diabetes as a confounding factor to make its predictions.

## 2. Materials and Methods

### 2.1. Dataset and Label Distribution

The data used in this study were acquired from the King Abdullah International Medical Research Center (KAIMRC)’s big ocular images dataset [43]. The collection of these images was approved by the Institutional Review Board (IRB) of the Ministry of National Guard Health Affairs under the protocol number RC-19-316-R. As the images were fully anonymized and collected retrospectively, the requirement for obtaining informed consent was waived by the IRB. Additionally, this study adhered to the principles outlined in the Declaration of Helsinki.

We used 5016 fundus images with dimensions of 2576 × 1934 pixels collected retrospectively from patients using two different optical coherence tomography (OCT) machines (DRI OCT Triton and 3D OCT-2000; Topcon, Tokyo, Japan).

The OCT machines were set to the macular fixation position, utilizing either radial or 3D scan patterns. All images were captured in color image type with a 45° angle, utilizing an operating distance of 34.8 mm for the DRI OCT Triton and 40.7 mm for the 3D OCT-2000. The flash level was typically set to 2, and the illumination level to 4; however, the flash and illumination levels were adjustable according to the patient’s condition. All images were stored in JPG format.

Each image was labeled using its demographic attributes, including age, sex, diabetes status, fundus photographs, and a binary classification of HTN status (HTN or non-HTN). The latter classification was based on the patient’s history of having HTN and/or at least three readings of high BP and/or undergoing antihypertensive treatment. These data were extracted from their electronic medical record progress notes written by an ophthalmologist on the date the fundus image was captured.

The dataset was divided into training, validation, and test sets. Approximately 60% of the data were allocated to model training, 20% to model selection and hyperparameter tuning, and the remaining 20% to testing. The dataset splits were made such that the three subsets contained the same ratios of HTN to non-HTN patients. Additionally, we ensured that the ratio of HTN to diabetic patients was consistent across all subsets. To ensure that no patient appeared in multiple subsets, a patient-specific split was performed in which patients with both right- and left-eye images were allocated to either the training, validation, or test set. This approach ensured that each patient was included in only one subset.

#### 2.1.1. Descriptive Analysis

The average age of the patients, identified through a descriptive analysis of the dataset, was 58.65 ± 22.37 years. The HTN and non-HTN datasets had average ages of 62.48 ± 10.23 and 53.25 ± 31.77 years, respectively. The dataset comprised 44% males (n = 2224) and 56% females (n = 2792). Moreover, there was a correlation between HTN and diabetes with 96% of patients with HTN also diagnosed with diabetes, compared to only 64% of non-HTN patients. Further details of the dataset characteristics are provided in Table 1.

#### 2.1.2. Data Preprocessing and Augmentation

Images were cropped and resized to 512 × 512 pixels, normalized to the range (0, 1) using min–max normalization and standardized using the z-score formula with the mean and standard deviation of ImageNet. Age was standardized around a mean of zero with a unit standard deviation. Standardization was applied to each subset independently, ensuring that there was no data leakage.

Additionally, we used standard image augmentation techniques to prevent model overfitting owing to the limited amount of training data. Specifically, we performed rotation, flipping, and blurring, which are commonly used to augment data for training DL models. The images were randomly rotated up to 360° and flipped horizontally. Additionally, a random Gaussian blur with a kernel size of three was applied to further introduce small variations into the dataset.

### 2.2. Classification Models

Four MMDL systems were developed to classify HTN occurrences by integrating fundus photographs and demographic features using intermediate and late fusion techniques. Detailed descriptions of each system are provided in Section 2.2.1. These four systems comprised three primary neural network components: “FundusPath” to process fundus photographs, “DemographicPath” to handle age and gender features, and “FusionPath” to integrate features from both modalities. A diagram of the four systems is presented in Supplementary Figure S2.

For FundusPath, we employed RETFound, a foundation model pretrained on 1.6 million retinal images from various sources [44]. For DemographicPath, we utilized a fully connected neural network (FCNN). Additionally, leaky rectified linear activation functions (leaky ReLUs) [45] were used and dropout layers were added [46]. Like DemographicPath, FusionPath is also an FCNN that received the outputs from DemopgrahicPath and FundusPath to generate an HTN prediction. Additionally, we evaluated four separate unimodal systems for processing either fundus photographs or demographic features alone for a comparison with the four MMDL systems, as described in Section 2.2.2.

#### 2.2.1. MMDL Systems

This section describes the four MMDL systems employed in this study: IntermediateFusion, PredictionFusion, LateFusion, and VotingFusion. These four systems utilized the three network paths described in Section 2.2 in various ways to make their predictions.

##### IntermediateFusion and PredictionFusion

**Figure 1:**
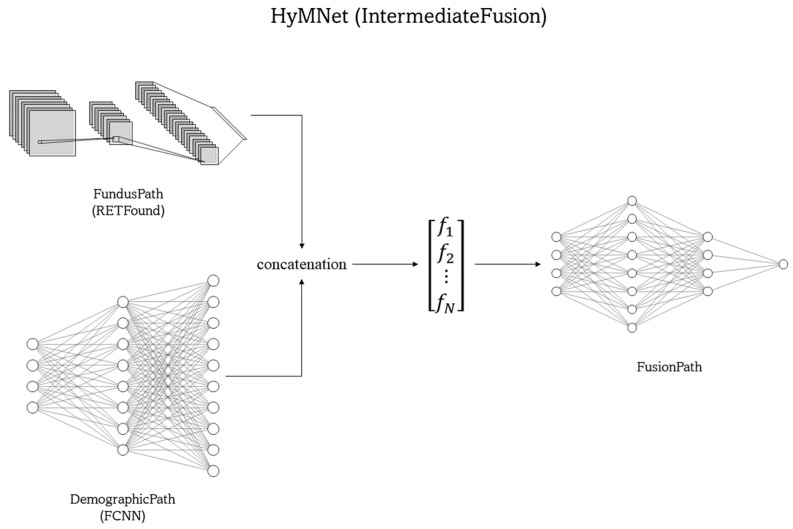
IntermediateFusion system diagram. Deep feature outputs from FundusPath and DemographicPath, represented as $f_{1}-f_{N}$, were concatenated and fed into FusionPath, and its output was used to update the trainable parameters of the three networks.

As shown in Figure 1, in the IntermediateFusion system, FundusPath extracted eight features from the fundus photographs, whereas DemographicPath output 32 deep features from the demographic information. The feature vectors generated by both networks were then concatenated to form a composite feature representation, which was subsequently fed into the FusionPath network for HTN classification. This intermediate fusion system is referred to as HyMNet.

In the PredictionFusion system, fundus images and age and sex characteristics were passed into FundusPath and DemographicPath, respectively. However, unlike feature fusion, each of these components produced a single output, which we refer to as the prediction logit. Consequently, the prediction logits, rather than the deep features like in IntermediateFusion, from both paths were concatenated and passed into FusionPath.

In both systems, the three networks (FundusPath, DemographicPath, and FusionPath) were trained jointly.

##### LateFusion

This system combined the prediction logits obtained from a RETFound model with age and sex features. The concatenated data were then fed into a classifier to make the final HTN prediction. We evaluated three different classifiers: extreme gradient boosting (XGBoost), support vector machine (SVM), and FCNN.

##### VotingFusion

For the VotingFusion system, we derived prediction logits from the fundus photographs using a trained RETFound model and demographic features using a trained FCNN. Additionally, we acquired a third prediction logit from a fully trained IntermediateFusion system. These three prediction logits were then concatenated and fed into the three classifiers. Furthermore, we assessed an ensemble technique for the three prediction logits using soft voting, where the average of the three individual predictions was considered the final prediction.

#### 2.2.2. Unimodal Systems

This study also compared the effectiveness of multimodal and unimodal HTN detection models. Therefore, the performance of RETFound, a unimodal model, was evaluated on the fundus photographs. Additionally, we constructed a model using demographic features (age and gender) with the three classifiers: DemographicXGB, DemographicSVM, and DemographicFCNN.

### 2.3. Training Configurations

We used the PyTorch framework for the DL pipeline [47], whereas SVM modeling, data-handling procedures, and vectorized operations were facilitated using scikit-learn, Pandas, and NumPy libraries, respectively [48,49,50]. Additionally, the XGBoost library was used to implement the XGBoost classifier [51]. During the neural network training, we used a binary cross-entropy loss function and the AdamW optimizer [52]. All training processes were conducted with a batch size of 16. Additionally, 25 training epochs were used for validation runs to save computational costs, while we used 50 epochs for testing all the systems, except for the FCNN models, for which 250 epochs were used. The optimal checkpoints for all the epochs were selected. Furthermore, we employed a cosine scheduler to decrease the learning rate after each iteration.

### 2.4. Model Selection and Hyperparameter Tuning

To determine the optimal neural network architecture and hyperparameters for the main components of the MMDL systems, we conducted several experiments on the validation set using the AUC score as the performance metric.

To select the best architecture for FundusPath, we tested various CNN and vision transformer architectures, including ResNet50 and DenseNet-201 pretrained on ImageNet1k, DINOv2 pretrained on LVD-142M [53,54,55,56,57,58], and RETFound pretrained on 1.6 million retinal images [44], with different learning rates. Based on the results, we selected the best performing model. The results for each network are presented in Table S3 of the Supplementary Materials.

For DemographicPath and FusionPath, we experimented with various numbers of layers and learning rates. We also used cross-validation techniques to conduct hyperparameter tuning to select the optimal XGBoost and SVM parameters for each system.

Additionally, as described in Section S1.2 of the Supplementary Materials, we studied the effect of fundus image size and concluded that increasing the size from 224 × 224 pixels to 512 × 512 pixels slightly improved the performance. Moreover, using the IntermediateFusion system, we further examined the effect of the feature vector size from both FundusPath and DemographicPath and present our findings in Section S1 of the Supplementary Materials. A decision threshold of 0.5 was used to generate the classifications of the HTN performance metrics.

### 2.5. Statistical Analysis

Nonparametric bootstrapping was employed to assess the statistical significance of our results. This approach involved resampling the test set with replacement data, where the number of samples obtained was equal to the total number of observations in the test set. By evaluating the performance of the model on these resampled datasets, we obtained insights into its true capabilities.

To obtain a robust estimate of the model performance, we repeated this resampling procedure 10,000 times to balance the computational efficiency and accurate parameter estimation. We computed the performance metrics, such as the AUC and area under the precision–recall curve (AUPRC) for each iteration. To determine the 95% confidence intervals (CIs) for these metrics, we reported the values at the 2.5th and 97.5th percentiles. This approach provided a range within which we could expect the true performance of the model to decrease, accounting for data variability and the potential impact of sampling bias.

### 2.6. Experimental Environment

All experiments were conducted on a workstation equipped with an AMD Ryzen Threadripper PRO 5955WX 16-Core processor, an NVIDIA RTX A6000 GPU, and 64 GB RAM.

## 3. Results

This section begins by presenting the results for the main experiments proposed, comparing HyMNet with unimodal systems for HTN classification. Then, in Section 3.1, the influence of diabetes on HTN detection capabilities was analyzed. Finally, Section 3.2 shows a region of interest visualization that depicts the features RETFound uses for predicting hypertension.

**Figure 2:**
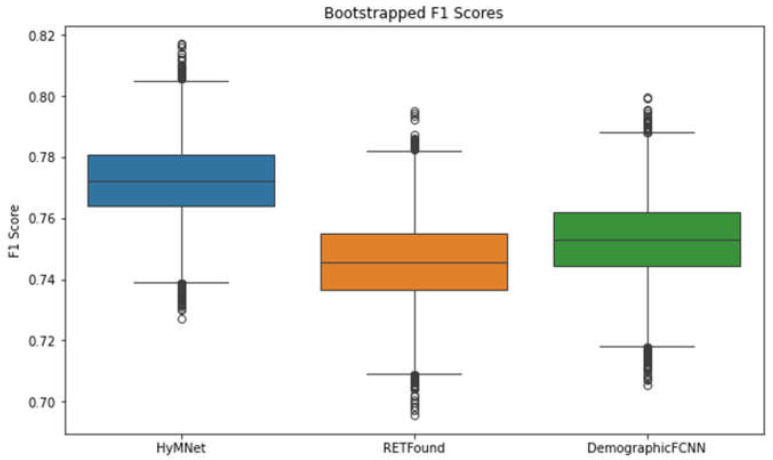
Box and whisker plots of the F1 scores of HyMNet, RETFound, and DemographicFCNN. The performance increase achieved by incorporating demographic features with fundus photographs is evident. These plots were generated using 10,000 bootstrapped F1 scores.

Table 2 presents the results of the proposed HyMNet and the unimodal RETFound and DemographicFCNN models. Notably, HyMNet obtained the highest scores across all five metrics, with an F1 score of 0.771 [0.747, 0.796] compared to 0.745 [0.719, 0.772] and 0.752 [0.727, 0.778] of RETFound and DemographicFCNN, respectively. Figure 2 shows the F1 scores of the three systems on a box and whisker plot, which underscores the significance of including demographic features in conjunction with fundus photographs for improving the performance of HTN prediction models.

To evaluate the statistical significance of the F1 score of HyMNet compared with other systems, we employed the method used in [59]. Specifically, we computed the difference between the bootstrapped F1 scores of HyMNet and RETFound using an identical bootstrapped sample. Subsequently, we ascertained whether the 95% CI for this difference was zero or negative. We discovered that the difference of 0.02 [0.003, 0.038] between the F1 scores excluded zero, leading us to conclude that the performance of HyMNet was significantly better than that of RETFound. We repeated this experiment for the DemographicFCNN and found that the performance increase was not statistically significant, with a difference of 0.013 [−0.005, 0.032] between the F1 scores.

Additional results for all systems used in this study are presented in Supplementary Table S2. Evidently, combining HyMNet with RETFound and DemographicFCNN in the VotingFusionEnsemble method further increased the performance slightly across all metrics, except recall.

**Figure 3:**
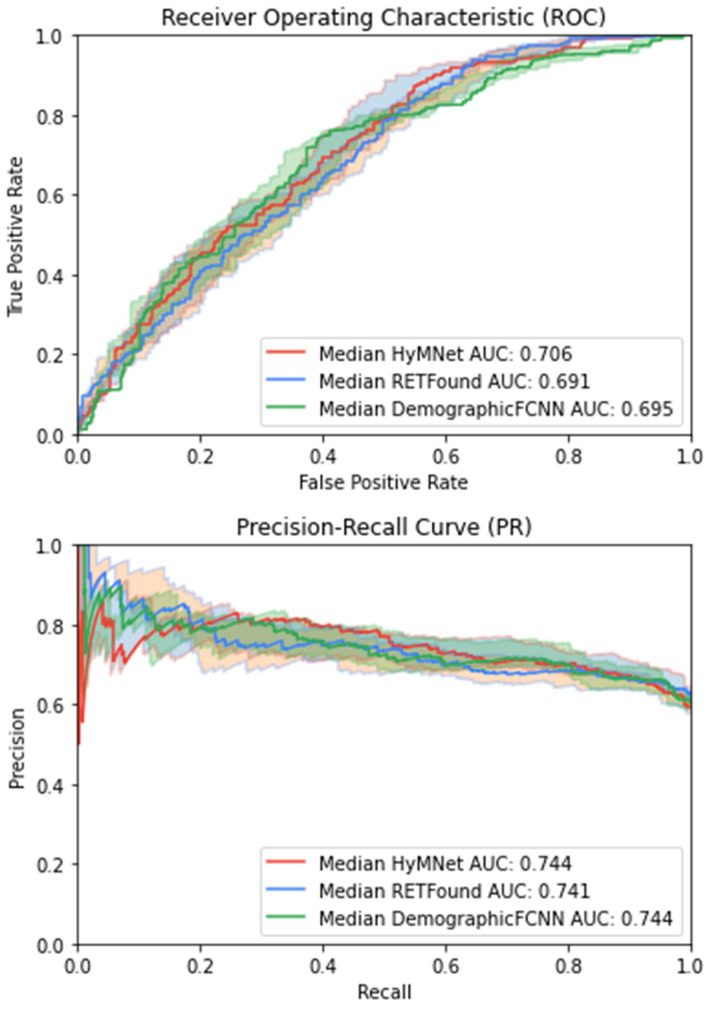
ROC and AUPR curves of multimodal and unimodal systems. The diagrams were generated using the median AUC score predictions for the 10,000 bootstrapped runs. The 97.5th and the 2.5th percentiles of the ROC and AUPR curves are represented by lighter colors.

Figure 3 shows the ROC and AUPR curves for HyMNet, RETFound, and DemographicFCNN. Specifically, we plotted the bootstrapped run with the median AUC and AUPRC values (presented in dark colors) and used the 97.5th and 2.5th percentiles from the 10,000 bootstrapped results to plot the intervals (highlighted in lighter colors). A large variability can be observed in both plots.

### 3.1. Influence of Diabetes on HTN Detection

We also examined the effect of diabetes on HTN detection. Table 3 presents the performance results of HyMNet for patients with and without diabetes. The F1 and AUPRC scores were higher by nearly 0.5 for patients with diabetes, suggesting that diabetes was a confounding variable for HTN prediction. However, it is important to note that the distribution of positive and negative patients here was unbalanced, following the original distribution for the entire dataset shown in Table 1.

### 3.2. Region of Interest Visualization

**Figure 4:**
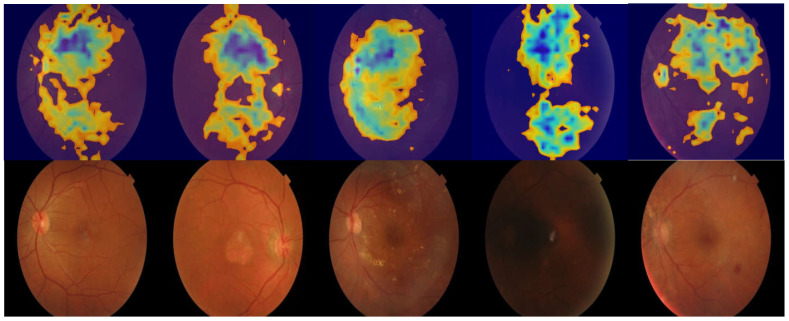
Grad-CAM for fundus photographs with HTN. We used RETFound for this analysis.

We employed gradient-weighted class activation mapping [60] to visualize the regions of interest used by RETFound for HTN predictions. Figure 4 shows a heat map, wherein the prediction influence begins with the most elevated area in the retina representing the red region (e.g., blood vessels, disc margin, and retinal thickening) and then goes down through the orange and yellow regions until it reaches the lowest area represented in blue (e.g., retinal thinning).

## 4. Discussion

HTN can have various effects on the vascular system, which can be visualized through fundus photographs. However, these effects may occur at the microvascular level, which are challenging for human observers to detect, particularly during the early stages. DL techniques offer a solution to this challenge owing to their high sensitivity to such changes, potentially enabling the early detection of HTN patients and preventing the onset of severe health conditions. Previous studies have shown that age and sex are strong predictors of HTN [61], with males being at a higher risk of developing it at an earlier age than females [62]. Based on these findings, it is natural to explore the integration of readily available and robust sociodemographic predictors, such as age and sex, for developing MMDL models for HTN prediction from fundus photographs. This approach can result in an effective and efficient model for the early detection and management of HTN, ultimately improving patient outcomes.

Recently, MMDL has emerged as a promising research area in the field of medical AI [63]. By utilizing diverse heterogeneous data, MMDL systems attempt to mimic the decision-making processes of medical experts, which are typically based on various sources. Qiu et al. [64] showed that the predictive performance for dementia and Alzheimer’s disease was improved using DL models on magnetic resonance imaging scans integrated with demographic features and EHRs. Additionally, Lee et al. [65] demonstrated an increased CVD risk prediction performance using an MMDL system by combining fundus photographs with cardiometabolic risk factors.

In this study, we conducted several experiments using the proposed HyMNet model, which integrates age and sex information with fundus images, and it outperformed the RETFound and DemographicFCNN models with an F1 score of 0.77 for HTN prediction. This improved performance can be attributed to the fact that HTN typically develops later in life and may exhibit different onset patterns in males and females [62]. Our unimodal fundus model for HTN prediction achieved an AUC score of 0.69, which was within the range of values reported in previous studies [40,62].

### Limitations and Future Research Directions

The primary limitation of this study was the absence of HTN stages in the dataset. Specifically, we were unable to assess the capability of our model for predicting the different stages of HTN because we did not have information on the HTN stage for each patient. Similarly, we were unable to measure the effect of hypertensive retinopathy on the ability to predict systemic HTN and believe that this can be a promising direction for future research.

This study was also limited by a small dataset, which led to unstable bootstrapping performance, as indicated by variations of ±0.03 in the 95% CI from the mean. Therefore, larger datasets would allow for a more conclusive comparison of whether adding age and sex features enhances the system’s ability to predict HTN from fundus photographs and to what extent.

To expand the proposed MMDL framework, future studies could integrate vessel segmentation methodologies, similar to Dai et al. [66], who achieved a higher AUC with their CNN model by using a “segmented dataset” composed only of retinal blood vessels extracted using a pretrained U-Net-based model. Furthermore, multiclass classification problems can be investigated to gauge the ability of DL models to detect the HTN stage using fundus images and demographic features. Models that encompass HTN patients at various stages can potentially uncover valuable insights regarding their capacity to detect HTN during its early stages as well as the areas of the image that are instrumental in generating such predictions.

Another limitation is the lack of diversity in the dataset. Including data from multiple races and underlying conditions could increase the generalizability of the model. Furthermore, to facilitate the integration of the proposed system into clinical environments, it is imperative to obtain additional data from multiple healthcare institutions, which can enhance the generalizability and overall performance of the system.

## 5. Conclusions

In this study, we assessed four MMDL configurations and compared their performances with established benchmarks using 5016 fundus photographs. We also trained the RETFound unimodal system using only fundus photographs. By incorporating age and sex variables, the proposed HyMNet model obtained better performance (F1 score 0.77) than the RETFound unimodal system (F1 score 0.74). Furthermore, we examined the effect of diabetes on HTN detection by measuring the performance results of HyMNet for patients with and without diabetes. The F1 and AUPRC scores were higher by nearly 0.5 for patients with diabetes. Our results suggested that HTN can be better examined with MMDL systems than unimodal systems and diabetes is a confounding variable for HTN prediction. There is significant potential in the future for MMDL systems to enhance HTN detection by utilizing public fundus photographs and incorporating other cardiometabolic risk factors.

## Figures and Tables

**Table 1:** Dataset characteristics.

	Total (n = 5016)	HTN (n = 2937)	Non-HTN (n = 2079)
Age (years), mean ± SD	58.65 ± 22.37	62.48 ± 10.23	53.25 ± 31.77
Gender, n (%)			
Male	2224 (44%)	1294 (44%)	930 (45%)
Female	2792 (56%)	1643 (56%)	1149 (55%)
Diabetes status, n (%)			
Positive	4145 (83%)	2817 (96%)	1328 (64%)
Negative	871 (17%)	120 (4%)	751 (36%)

**Table 2:** Performance results of multimodal and unimodal systems. The results include the 95% CI generated from the bootstrapping technique mentioned in Section 2.5. A classification threshold of 0.5 was used for the F1 score, precision, recall, and specificity.

Model	F1 Score	AUC	PR	Accuracy	Precision	Recall
HyMNet	0.771 [0.747, 0.796]	0.705 [0.672, 0.738]	0.743 [0.703, 0.784]	0.690 [0.662, 0.719]	0.683 [0.65, 0.716]	0.887 [0.862, 0.912]
RETFound	0.745 [0.719, 0.772]	0.690 [0.657, 0.724]	0.740 [0.701, 0.78]	0.682 [0.647, 0.717]	0.668 [0.639, 0.698]	0.821 [0.791, 0.852]
DemographicFCNN	0.752 [0.727, 0.778]	0.694 [0.661, 0.727]	0.742 [0.703, 0.782]	0.661 [0.632, 0.69]	0.662 [0.63, 0.695]	0.871 [0.845, 0.898]

**Table 3:** Effect of diabetes on HTN detection. The table presents the results of HyMNet or HTN-detection in patients with and without diabetes. The CI represents the 95% CI generated from the bootstrapping technique mentioned in Section 2.5. A classification threshold of 0.5 was used for the F1 score, precision, recall, and specificity.

Diabetes Status	F1	AUC	AUPRC	Accuracy	Precision	Recall
Positive	0.796 [0.772, 0.821]	0.68 [0.642, 0.717]	0.788 [0.748, 0.828]	0.696 [0.665, 0.727]	0.716 [0.684, 0.749]	0.895 [0.869, 0.921]
Negative	0.466 [0.352, 0.581]	0.704 [0.617, 0.79]	0.306 [0.202, 0.411]	0.642 [0.57, 0.715]	0.344 [0.237, 0.451]	0.78 [0.636, 0.923]

## Data Availability

The data presented in this study are available on request from the following link: https://kaimrc.ksau-hs.edu.sa/En/Pages/Ocular.aspx (accessed on 15 October 2024). The code and model weights of the proposed system are available at https://github.com/MohammedSB/HyMNet (accessed on 15 October 2024).

## Supplementary Materials

The following supporting information can be downloaded at: https://www.mdpi.com/article/10.3390/bioengineering11111080/s1, Figure S1: Feature embedding matrix; Figure S2: Multimodal systems diagram; Table S1: We report the results for hypertension classification for linear probing and fine-tuning two CNN and ViT models, pre-trained on natural datasets; Table S2: We evaluate the image size has on the ability to classify hypertension. Increasing the image size from 224 × 224 to 512 × 512 results in slightly better performance; Table S3: Multimodal and unimodal systems results.

## Abbreviations

AI	Artificial intelligence
AUC	Area under the ROC curve
BP	Blood pressure
CVD	Cardiovascular disease
DL	Deep learning
FCNN	Fully connected neural network
HTN	Hypertension
KAIMRC	King Abdullah International Medical Research Center
MAE	Mean absolute error
MMDL	Multimodal deep learning
OCT	Optical coherence tomography
AUPRC	Area under the precision–recall curve
ReLU	Rectified linear unit
ROC	Receiver operating characteristic
SVM	Support vector machine
XGBoost	Extreme gradient boosting
