# Supplementary material for: HyMNet: A Multimodal Deep Learning System for Hypertension Prediction Using Fundus Images and Cardiometabolic Risk Factors
Source: Bioengineering (Basel). 2024 Oct 29;11(11):1080. doi: 10.3390/bioengineering11111080 (PMC11591283; doi:10.3390/bioengineering11111080)
Supplement: Supplementary file 1 [file bioengineering-11-01080-s001.zip › bioengineering-3242403-supplementary.pdf]

HyMNet: a Multimodal Deep Learning System for Hypertension Classification using Fundus Photographs and Cardiometabolic Risk Factors

Supplementary Results

S1. Ablation Study

S1.1 Model Selection

We evaluated four models for the fundus path, two of which were CNN models and two were Vision Transformers. Specifically, we tested ResNet50 and DenseNet201 pre-trained on ImageNet1k, DINOv2 ViT-L/14 pre-trained on LVD-142M and RETFound pre-trained on 1.6 million retinal images from various sources. For each model, we tuned the learning rate for the newly initialized linear layer with the values {1e-2, 5e-2, 1e-3, 5e-3} and learning rates {1e-4, 5e-4, 1e-5, 5e-5, 1e-6, 5e-6} for the pre-trained backbone. In Supplementary Table 1, we present the AUC for linear-probing and fine-tuning for the best-performing checkpoint across the learning rates. We used 25 epochs training epochs for each run.

Table S1. We report the results for hypertension classification for linear probing and fine-tuning two CNN and ViT models, pre-trained on natural datasets. Only the AUC for the best performing hyperparameter and checkpoint is shown.

Model	Linear Probing	Fine-Tuning
ResNet50	0.650	0.690
DenseNet201	0.643	0.677
DINOv2	0.655	0.676
RETFound	**0.685**	**0.695**

S1.2 Effect of Image Size

In this section, we study the effect of image size on hypertension prediction ability. We evaluate the difference between processing fundus photographs at sizes 224x224 and 512x512 using RETFound on the test set. We observe a slight performance increase across F1, AUC, PR, and Accuracy scores, and a relatively larger increase for Recall. For this reason, we continued to use image size 512x512 for processing fundus photographs.

Table S2. We evaluate the image size has on the ability to classify hypertension. Increasing the image size from 224x224 to 512x512 results in slightly better performance.

Image Size	F1	AUC	PR	Accuracy	Precision	Recall
224	0.73	0.70	0.8	0.65	**0.68**	0.79
512	**0.75**	**0.71**	**0.81**	**0.67**	**0.68**	**0.84**

S1.3 Joint Fusion Feature Vector Size

We attempt to find the optimal sizes for the fundus and demographic paths in the joint fusion architecture. We scan {8, 32, 128} feature vector sizes for both paths, and report our

results in Supplementary Figure 1. For the fundus path, we experimented with using the patch embeddings ("Patch") of the vision transformer, without an added trainable linear layer, as an output. Our experiments conclude that using a feature vector size of 8 for the fundus path and a size of 32 for the demographic best performs the best. We use RETFound for the fundus path.

**Fundus** / **Demo**	**Patch**	**8**	**32**	**128**
**8**	0.658	0.647	0.643	0.659
**32**	0.648	0.67	0.65	0.664
**128**	0.603	0.664	0.657	0.649

Figure S1. Feature embedding matrix. We report the AUC for using different feature vector sizes for the fundus path "Fundus" and demographic path "Demo." As show in the figure, an embedding of size 8 for the fundus path and size 32 for the demographic path results in the optimal AUC.

Figure S2.

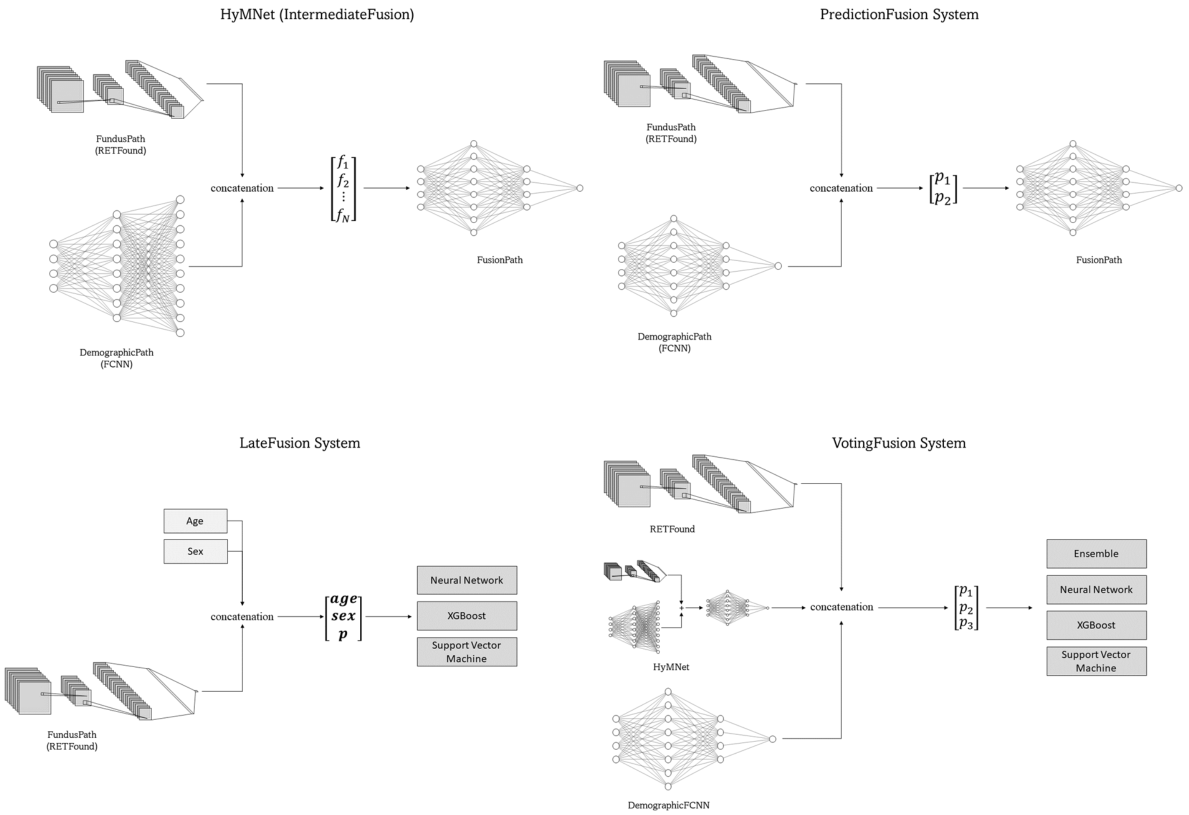

Figure S2. Multimodal systems diagram. On the top left, the IntermediateFusion system is shown, where deep feature outputs from the FundusPath and the DemographicPath, represented as $f_1 - f_N$, are concatenated and fed into the FusionPath. The top right diagram shows the PredictionFusion system, where predictions logits, p_1 and p_2, are concatenated instead. The bottom two diagrams show the LateFusion and VotingFusion systems. In LateFusion, age and gender features are concatenated to the prediction logit of a fully trained FundusClassifierPath and passed into three different classifiers. Lastly, in VotingFusion, the prediction logit of a fully trained FeatureFusion system is concatenated with the prediction logits of a trained FundusClassifierPath and a trained DemographicClassifierPath.

Table S3.

Table S3. Multimodal and unimodal systems results. The table presents the F1-score, AUC, PR, precision, recall, and specificity scores for all multimodal and unimodal systems used. CI represents the 95% confidence interval generated from the bootstrap technique mentioned in Section 2.5. A classification threshold of 0.5 was used for the F1-score, precision, recall, and specificity.

Model	F1	AUC	PR	Accuracy	Precision	Recall
Multimodal Systems						
HyMNet (IntermediateFusion)	0.771 [0.747, 0.796]	0.705 [0.672, 0.738]	0.743 [0.703, 0.784]	0.690 [0.662, 0.719]	0.683 [0.65, 0.716]	0.887 [0.862, 0.912]
PredictionFusion	0.758 [0.733, 0.784]	0.672 [0.637, 0.707]	0.713 [0.671, 0.755]	0.679 [0.646, 0.713]	0.676 [0.647, 0.706]	0.860 [0.833, 0.888]
LateFusionXGB	0.758 [0.733, 0.783]	**0.717 [0.684, 0.75]**	0.749 [0.708, 0.791]	0.685 [0.652, 0.719]	0.68 [0.652, 0.708]	0.848 [0.819, 0.877]
LateFusionSVM	0.768 [0.744, 0.792]	0.685 [0.652, 0.718]	0.732 [0.692, 0.773]	0.657 [0.625, 0.689]	0.67 [0.641, 0.699]	0.925 [0.905, 0.946]
LateFusionFCNN	0.751 [0.726, 0.777]	0.697 [0.664, 0.73]	0.742 [0.703, 0.782]	0.682 [0.649, 0.715]	0.673 [0.644, 0.702]	0.836 [0.807, 0.866]
VotingFusionXGB	0.745 [0.718, 0.772]	0.704 [0.671, 0.737]	0.751 [0.712, 0.79]	0.680 [0.646, 0.715]	0.667 [0.639, 0.696]	0.823 [0.792, 0.854]
VotingFusionSVM	0.770 [0.747, 0.794]	0.696 [0.663, 0.73]	0.746 [0.707, 0.786]	0.644 [0.613, 0.675]	0.662 [0.634, 0.691]	**0.960 [0.945, 0.976]**
VotingFusionFCNN	0.739 [0.713, 0.766]	0.682 [0.648, 0.716]	0.735 [0.696, 0.775]	0.674 [0.64, 0.708]	0.659 [0.63, 0.688]	0.82 [0.789, 0.851]
VotingFusionEnsemble	**0.772 [0.748, 0.796]**	0.712 [0.68, 0.744]	0.748 [0.708, 0.788]	**0.692 [0.664, 0.72]**	**0.688 [0.655, 0.721]**	0.881 [0.856, 0.906]
Unimodal Systems						
RETFound	0.745 [0.719, 0.772]	0.690 [0.657, 0.724]	0.740 [0.701, 0.78]	0.682 [0.647, 0.717]	0.668 [0.639, 0.698]	0.821 [0.791, 0.852]
DemographicXGB	0.756 [0.73, 0.782]	0.697 [0.665, 0.73]	0.736 [0.695, 0.778]	0.684 [0.655, 0.713]	0.696 [0.662, 0.73]	0.827 [0.797, 0.858]
DemographicSVM	0.765 [0.74, 0.79]	0.706 [0.674, 0.738]	**0.752 [0.713, 0.791]**	0.671 [0.642, 0.7]	0.661 [0.629, 0.695]	0.907 [0.884, 0.93]
DemographicFCNN	0.752 [0.727, 0.778]	0.694 [0.661, 0.727]	0.742 [0.703, 0.782]	0.661 [0.632, 0.69]	0.662 [0.63, 0.695]	0.871 [0.845, 0.898]
